# Application of the inverted classroom model in the teaching module “new classification of periodontal and peri-implant diseases and conditions” during the COVID-19 pandemic

**DOI:** 10.3205/zma001485

**Published:** 2021-06-15

**Authors:** Marius Crome, Knut Adam, Marco Flohr, Alexander Rahman, Ingmar Staufenbiel

**Affiliations:** 1 Hannover Medical School, Department of Conservative Dentistry, Periodontology and Preventive Dentistry, Hannover, Germany

**Keywords:** case-based learning, student-centered, inverted classroom model, dental education, COVID-19, new classification of periodontal diseases, periodontitis

## Abstract

**Background:** Due to the need for patient-free dental education during the COVID-19 pandemic, Hannover Medical School (MHH) implemented a new periodontology module. Its didactic structure was based on the “inverted classroom model” (ICM) in combination with elements of case-based learning. The educational objective was to increase the diagnostic confidence of dental students in the classification of periodontal patients (staging & grading), based on 33 digitized patient cases. To assess the suitability of the module for future dental curricula, this study aimed to evaluate student satisfaction and skills acquisition.

**Methods: **The periodontology module, which was attended by final year dental students of MHH (n=55, mean age: 26.5±3.9 years, male/female ratio: 24.1%/75.9%) was evaluated in a two-tiered way. Student satisfaction was recorded using a questionnaire. Learning success was assessed by comparing error rates in patient case classifications before (T_0_) and after (T_1_) participation in the periodontology module.

**Results:** The study found a high level of student satisfaction with the ICM format and a significant reduction in error rates (T_0_ error rate=28.3%; MV±SD=3.12±1.67 vs. T_1_ error rate=18.7%; MV±SD=2.06±1.81; Δ=9.6%). However, of the 11 diagnostic decisions required, only four parameters (extent, grading, percentage of bone loss per age, phenotype) showed significant improvements, with effect sizes ranging from small to medium.

**Conclusions: **The ICM-based teaching concept is definitely not an alternative to patient-based learning. However, in regard to student satisfaction and learning success, it might be superior to conventional classroom-based lectures, especially when complex topics are covered. In summary, the newly developed periodontology module may be a useful addition to traditional dental education in future curricula, even for the time after the COVID-19 pandemic.

## Zusammenfassung

**Hintergrund:** Im Zuge der Patienten-freien Lehre zur Zeit der COVID-19-Pandemie wurde ein auf dem „Inverted Classroom Model“ (ICM) basierendes Parodontologie-Modul in die zahnmedizinische Lehre eingeführt. Anhand von 33 digital ausgearbeiteten Patientenfällen sollte die diagnostische Sicherheit von angehenden Zahnärztinnen und Zahnärzten bei der Klassifizierung parodontal erkrankter Patienten (Staging & Grading) gesteigert werden. Um die Eignung des Moduls auch hinsichtlich der Entwicklung zukünftiger, zahnmedizinischer Curricula überprüfen zu können, war es das Ziel dieser Studie, die Zufriedenheit und den Kompetenzerwerb der Studierenden zu ermitteln.

**Methodik: **Die Evaluation des Parodontologie-Moduls, das von Zahnmedizinstudierenden der Medizinischen Hochschule Hannover aus dem 10. Semester (n=55, mittleres Alter: 26,5±3,9 Jahre, Verhältnis männlich/ weiblich: 23,6%/76,4%) besucht wurde, erfolgte zweigeteilt. So wurde die studentische Zufriedenheit Fragebogen-basiert erfasst. Der Lernerfolg wurde durch den statistischen Vergleich der Fehlerquote bei der Klassifizierung eines Patientenfalls vor (T_0_) und nach (T_1_) Teilnahme am Parodontologie-Modul ermittelt.

**Ergebnisse:** Es konnte eine hohe Zufriedenheit der Studierenden mit dem ICM-Format sowie eine signifikante Reduktion der Fehlerquote (T_0_ Fehlerquote=28,3%; MW±SA=3,12±1,67 vs. T_1_ Fehlerquote=18,7%; MW±SA=2,06±1,81; Δ=9,6%) festgestellt werden. Von den insgesamt 11 diagnostischen Entscheidungen zeigten aber nur vier Parameter (Ausdehnung, Grading, prozentualer Knochenabbau pro Alter, Phänotyp) eine signifikante Verbesserung mit schwach- bis mittelgradiger Effektstärke.

**Schlussfolgerung:** Das ICM-basierte Lehrkonzept stellt sicherlich keine Alternative zur Patienten-basierten Lehre dar, könnte aber hinsichtlich der Lehrzufriedenheit und des Lernerfolgs insbesondere bei komplexen Themengebieten der klassischen Präsenz-basierten Vorlesung überlegen sein. Das neu entwickelte Parodontologie-Modul stellt auch für die Zeit nach der COVID-19-Pandemie eine sinnvolle Ergänzung in der zahnmedizinischen Lehre dar.

## Summary

This article describes the integration of a new teaching format into dental education during the COVID-19 pandemic. The objective was to increase the confidence of 10^th^-semester students in the use of the new, recently published classification of periodontal diseases. The results showed very high student satisfaction and a significant increase in learning success.

## Background

**Figure 1:**
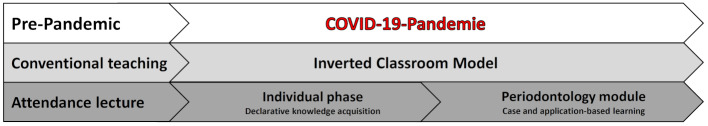
Teaching schedule for the 2020 summer semester

The COVID-19 pandemic poses an immense challenge to society in terms of solidarity and socioeconomics and is therefore negatively connoted. Conventional teaching methods and work processes cannot simply be maintained, but need to be adapted to the new situation. Both business and education are identifying ways and means to benefit from the pandemic-related adaptations in the long term [4], [19], [25], [35]. Against the background of infection control measures in the form of contact bans and social distancing, faster digitization in workplaces and educational institutions is definitely most important. As a result, the attendance culture prevalent in German businesses is increasingly being questioned in the light of the opportunities and synergies offered by telework or work from home, for instance [20]. This also applies to traditional, classroom-based dental and medical teaching, which is evidently not efficient enough. The German Council of Science and Humanities (Wissenschaftsrat), an advisory body to the Federal Government and State Governments, and Hochschulforum Digitalisierung, a think tank orchestrating the discourse on higher education in the digital age, have urged for restructuring measures, including the implementation of didactically valuable digital elements, since 2016 [10], [11], [18], [23], [34], [36]. And although Germany’s institutions of higher education considered themselves digitally well-equipped for the COVID-19 summer semester, according to a survey conducted by the Donors' Association for the Promotion of Humanities and Sciences (Stifterverband), many of them were only able to perform distance teaching based on mere information transfer without any underlying didactic concept, for reasons of infrastructure, staffing and time [21], [30]. In daily clinical routine, dental practitioners need to make numerous diagnostic and therapeutic decisions that substantially influence the course and success of a treatment. However, skills like “critical thinking” and “decision-making ability” have so far been taught only insufficiently in lecture-based education. Student-centered, didactically sound teaching formats would be necessary to properly prepare dental students for practice [3]. This is why the Hannover Medical School (MHH) Department of Conservative Dentistry, Periodontology and Preventive Dentistry developed a case-based teaching module, guided by the “inverted classroom model” (ICM). It was designed to compensate for the fact that students did not have the chance to improve their clinical decision-making skills by treating real patients [14]. The module aimed to ensure a deeper understanding and reliable clinical application of the new classification of periodontal diseases, published in 2018. This classification allows clinicians to combine a wide variety of diagnostic parameters in therapeutically and prognostically relevant categories and to develop treatment concepts tailored to patients’ individual needs [15], [29], [33]. As almost every second adult in Germany is affected by periodontitis and prevalence increases with age, profound knowledge of periodontology is a must for prospective dental practitioners [13]. The ICM, first used 20 years ago, is highly popular in the literature and considered one of the most important teaching concepts for higher education [2], [32]. Against the backdrop of new digital challenges, continuously growing scientific knowledge and lifelong learning needs, information processing is becoming more and more important to teaching than mere information transfer [34]. This is why the inverse principle was synergistically combined with “case-based learning” (CBL) for didactic reasons in this periodontology module (see figure 1 (Fig. 1)). Since this dental teaching concept was used for the first time at the MHH, our educational study aimed to assess student satisfaction and learning success. To evaluate the latter objective, the following hypothesis was formulated:

Participation in the periodontology module reduces students’ error rates in the classification of periodontal patients.

## Methods

### Participants

The participants in the periodontology module were MHH 10^th^-semester dental students, who acquired their knowledge of the new classification of periodontal diseases in a hybrid approach due to the pandemic. This approach included a conventional, classroom-based lecture course given in the 2019/2020 winter semester and an individualized and digitalized phase completed shortly before participation in the periodontology module. The classroom-based part consisted of five lectures of 45 minutes each, given on five days. The slides of the lectures and suggestions for further, in-depth reading for self-study were made available to the students via an e-learning platform (ILIAS; Integrated Learning, Information and Collaboration System; Peter L. Reichertz Institute). The students had already gained some practical experience in the treatment of periodontal patients in two clinical treatment courses (Conservative Dentistry Course I, 2018/2019 winter semester, Integrated Course I, 2019/2020 winter semester). In the 2020 summer semester, there were no clinical treatment courses due to the COVID-19 pandemic. 

#### Questionnaire

**Figure 2:**
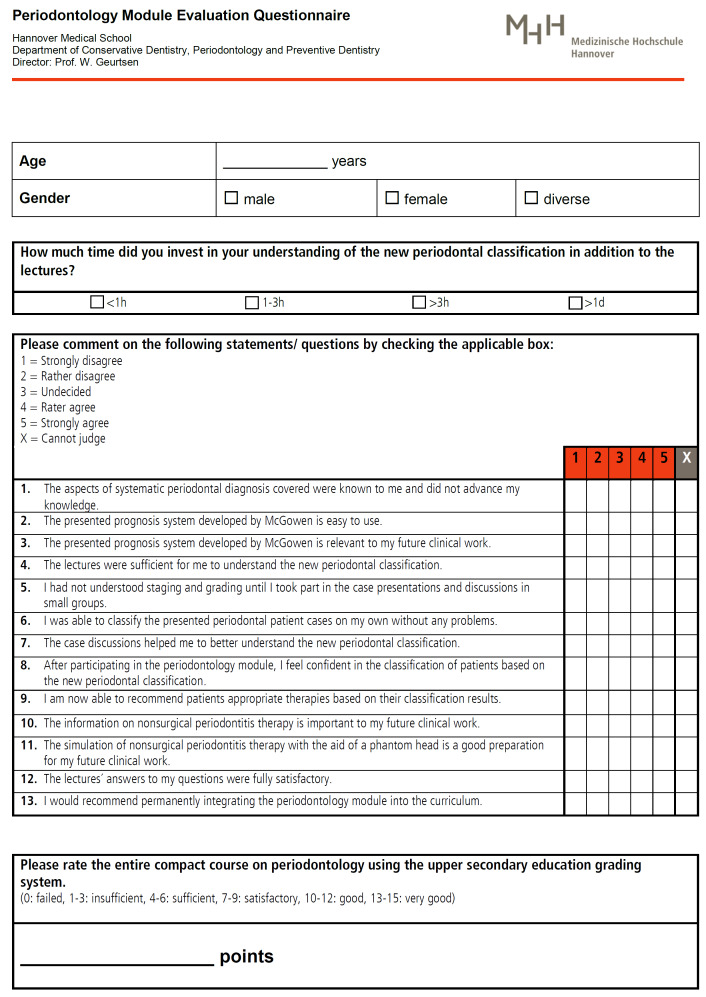
Evaluation sheet

To evaluate student satisfaction with the periodontology module, a 17-item questionnaire was prepared. Due to the short lead time, this tool could not be checked for reliability, so the data recorded were only analyzed descriptively. In addition to general questions (age, gender, time invested in self-study), the evaluation questionnaire included 13 Likert-type items with a 5-point scale (“strongly disagree”=1, “rather disagree”=2, “undecided”=3, “rather agree”=4, “strongly agree”=5, “cannot judge”=X, cf. figure 2 (Fig. 2)). Furthermore, the students rated the periodontology module using the German upper secondary education grading system (0: failed, 1-3: insufficient, 4-6: sufficient, 7-9: satisfactory, 10-12: good, 13-15: very good). 

#### Contents and scope of the periodontology module

The periodontology module comprised two teaching units of four hours each. At the start, each student received a comprehensive documentation of findings (dental findings, photo status, periodontal status, panoramic radiograph and/or x-ray status) in a real patient case for classification in accordance with the case definitions published in 2018 [33]. Most patient cases were generated on the basis of case presentations used in previous clinical treatment courses. It was taken into consideration that both dental findings and periodontal status might include mistakes or measuring errors. Correct classifications of the 33 patient cases included in the module were determined beforehand by three experienced periodontists. The students were expressly instructed to base their decisions on the radiographic images in case there were any inconsistencies in the documentations.

The classification of periodontitis consists of “staging” and “grading”. Staging (Stage 1, 2, 3, 4) aims to determine the severity and complexity of the disease and comprises five diagnostic aspects:

1. Interdental clinical attachment level in the region with the largest bone loss;
2. Radiographic bone loss in the region with the largest loss;
3. Tooth loss due to periodontitis,
4. Local complexity factors; and
5. Extent/distribution.

Grading (Grade A, B, C) aims to determine the progression rate of the disease and estimate the treatment result achievable on the basis of patient-specific factors. By definition, it comprises five subparameters:

1. Longitudinal comparison of radiographic bone loss;
2. Percentage of bone loss in the region with the largest loss divided by patient age;
3. Phenotype (periodontal destruction in relation to the presence of microbial biofilm);
4. Nicotine abuse; and
5. Glycohemoglobin concentration in the patient’s blood.

Since a longitudinal comparison of radiographic bone loss could not be reconstructed in all patient cases, this parameter was not included in the classification. So the 55 students used nine subparameters and the two main categories (staging & grading) to make a total of 11 diagnostic decisions per patient case. Subsequently, the students developed patient-specific treatment concepts, based on their diagnoses, and presented and discussed their concepts in small groups to comply with the pandemic-related hygiene rules. The lecturers (K.A. and I.S.) acted rather as mediators than as instructors, drawing the students’ attention to the critical points of the new classification. At the end of the case discussion phase, the students classified their patient cases again. 

#### Statistical analysis

The data collected were statistically analyzed using the IBM SPSS Statistics 26 software (IBM Corporation, Armonk, New York, USA), both in a descriptive way and with the aid of test methods based on inductive statistics. The Wilcoxon signed-rank test was used to investigate the total error rates of the students in the classification of patient cases before (T_0_) and after (T_1_) participation in the periodontology module. Besides, the chi-square test was used to analyze the stochastic independence of individual aspects of the new classification of periodontal diseases with regard to learning success.

## Results

From June 11^th^ to July 1^st^, 2020, a total of 55 MHH 10^th^-semester dental students participated in the periodontology module. Of these students, 76.4% (42/55) were female, 23.6% (13/55) male, and 0% diverse. The average age was 26.5±3.9 years.

### Analysis of the evaluation questionnaire

Basically, the periodontology module was rated “very good” by the students, with an average score of 13.66±1.04 out of 15 points. Moreover, they all agreed that this teaching concept should be permanently integrated into the dental curriculum (Questionnaire Item 13: “strongly agree”, n=52; “rather agree”, n=2). Although 81.5% of the students stated they were able to classify the patient cases by themselves without any problems (Item 6), 88.9% (n=48) said at the end of the periodontology module that they now felt more confident in the use of the new classification of periodontal diseases on patients (Item 8). The reasons they gave for this optimistic view were both the comprehensive case discussions, which had substantially contributed to a better understanding of the matter (Item 7: “strongly agree”, n=45; “rather agree”, n=9), and the lecturers’ fully satisfactory answers to questions arising from the discussions (Item 12: “strongly agree”, n=50; “rather agree”, n=3). As regards teaching and learning without the periodontology module, 42.6% of the participants stated that the classroom-based lectures alone would not have been sufficient for them to understand the new classification (Item 4). Besides, 79.6% of the students (n=43) answered that they had invested more than one day in self-study to prepare for the periodontology module. Still, 77.7% (n=42) indicated that they had not fully understood “staging and grading” until they took part in the subsequent attendance phase, i.e. the case discussions in small groups (Item 5).

#### Analysis of the learning success

In comparison with the ideal classifications defined by periodontal specialists, the students’ 605 diagnostic decisions included 168 errors in the first classification of their patient cases [T_0_ error rate: 27.8%; mean value (MV) ± standard deviation (SD): 3.07±1.56; parameters not specified: 19 (3.1%); n=55], and 106 errors [T_1_ error rate: 17.5%; MV±SD: 2.03±1.72; parameters not specified: 44 (7.2%); n=55] in the second classification (Δ=10.2%). Since an inductive statistical approach was used, the results of students who had not specified all parameters were eliminated; this reduced the sample size by 21 participants (n=34, T_0_ error rate: 28.3%; MV±SD: 3.12±1.67 vs. T_1_ error rate: 18.7%; MV±SD: 2.06±1.81; Δ=9.6%). The error rate proved to be significantly lower after participation in the periodontology module (asymptotic Wilcoxon test: z=-3.066; p=.002; n=34). Based on Cohen’s guidelines, the effect size may be considered medium (r=.52) [6]. Furthermore, detailed analysis of the individual diagnostic parameters using the chi-square test showed statistically significant correlations between participation in the periodontology module und reduced error rates only for three grading parameters and extent assessment (cf. table 1 (Tab. 1)):

- extent: Χ^2^=4.91, p=.027, Φ=-0.213, n=108;
- grading: Χ^2^=14.004, p<.001, Φ=-0.360, n=108;
- percentage of bone loss per age: Χ^2^=6.723, p=.01, Φ=-0.253, n=105;
- phenotype: Χ^2^=8.142, p=.004, Φ=-0.287, n=99.

Regarding effect size, however, it should be mentioned that only small to medium effects were achieved and seven diagnostic parameters did not show any significant changes at all.

## Discussion

This educational study aimed to assess an ICM-based periodontology module integrated into the dental curriculum on a short-term basis due to the COVID-19 pandemic in terms of student satisfaction and learning success. Statistical analysis showed that the participating students were satisfied with this teaching concept and supported its future use. Comparison of the students’ total error rates before and after participation in the periodontology module proved a significant learning effect. Basically, the positive results of this study are in line with other studies on comparable topics from the fields of medical and non-medical education [5], [9], [16], [22], [24], [31]. So it may be concluded that students prefer student-centered learning scenarios designed to improve higher cognitive skills (application of knowledge, analysis, synthesis and evaluation) to lecture-based knowledge transfer [1], [9], [27]. However, it should be ensured that the teaching materials made available beforehand are not too complex to be understood by the students on their own and that redundancies between the contents of self-study and classroom phases are avoided for reasons of efficiency [24]. If students cannot acquire the necessary declarative knowledge on their own in the self-study phase, due to motivation deficits or because the teaching materials are too complex or time-consuming, the use of the ICM will not make much sense. In this case, factual knowledge needs to be acquired in the classroom phase, even though certain skills that are important to professional life and lifelong learning (critical thinking, decision-making ability) are not improved in this way [8], [26], [32]. Considering these insights, the didactic structure of the periodontology module seems promising, since it allows students to efficiently work toward achieving their learning objective. It should be noted, however, that 79.6% of the students (n=43) stated they had invested more than one day in self-study. This substantial time investment suggests that the matter and the teaching materials may still be too extensive or complex, although the lecturers did their best to condense the contents of the primary literature used as much as possible. This assumption is confirmed by the students’ self-perception. Despite the distinct self-study phase, 77.7% said that they had not fully understood “staging and grading” until taking part in the case discussions of the periodontology module. This result is alarming, especially against the backdrop that the ICM project was integrated into the curriculum only as a substitute due to the COVID-19 pandemic and almost half the students did not consider the lectures, once the only teaching method used, sufficiently effective.

While assessments of student satisfaction with the ICM are relatively consistent in the literature, there is much less agreement on learning success. Researchers do agree that hybrid teaching concepts combining online and classroom elements have the potential to enhance learning success, as compared to traditional, single-phase concepts. To date, however, there has not been any strong evidence of the effectiveness of ICM concepts [5], [17]. In a systematic review from the field of dental education, only three of the five selected studies showed significant differences in learning effectiveness [9]. The results of a review by Hu et al. on the use of the ICM in nursing education, by contrast, were much more positive. The authors found the ICM to be superior to traditional teaching methods in eight out of nine studies [12]. The results of our study are similarly ambivalent with regard to learning success. The students made significantly fewer mistakes in the classification of patient cases after participating in the periodontology module (Δ=9.6%), but this does not prove an all-embracing learning effect, because only four of the 11 clinical/diagnostic parameters contributed to this reduction in the total error rate. Besides, the effect size was only small to medium, despite the considerable time and effort invested in learning. Detailed analysis of the error structure showed that especially for the parameters requiring transfer of knowledge and clinical experience (complexity, tooth loss) no improvement had been achieved. Parameters that are relatively easy to understand (extent, phenotype), by contrast, showed much better results. It is also interesting to see that the error rate for identifying the interdental space with the largest clinical attachment loss or radiographic bone loss remained unchanged. The case presentations revealed that it was difficult for the students to identify the outline of the crestal bone and, consequently, the interdental space with the largest radiographic bone loss in panoramic radiographs or x-ray statuses. This suggests that three-dimensional interpretation of radiographs is a skill that cannot be imparted by a single teaching unit but is acquired procedurally, as professional experience increases. Erroneous assessment of clinical attachment levels might also be explained by the fact that, in cases of inconsistencies in their documentations, the students did not base their decisions on the panoramic radiograph or x-ray status, but on the periodontal status, which had been determined in previous clinical treatment courses. Research has shown that clinicians’ experience substantially influences the accuracy of periodontal recordings and that measurements performed by students are significantly less accurate [7], [28]. In summary, the results of this educational study indicate that correct classification of periodontal patients requires a degree of clinical experience and procedural knowledge acquisition that the ICM format cannot adequately compensate for. When assessing the learning effect described in this study, it should be taken into account that the study design only followed a “before-after” approach, although a direct comparison of different didactic methods in a “cross-over” design would definitely have been more valuable. However, such a design could not be developed due to the short lead time and almost spontaneous implementation of the new teaching approach. It should also be considered that the questionnaire used to evaluate student satisfaction with the periodontology module has its limitations in terms of reliability and validity and can thus only show tendencies. The highly positive rating of the periodontology module (13.66 out of 15 points) is surely also attributable to the fact that the students welcomed it as a substitute for the clinical treatment of periodontal patients during the COVID-19 pandemic. 

## Conclusion

This educational study evaluated the implementation of an ICM-based periodontology module in dental teaching during the COVID-19 pandemic. The data collected are largely congruent with related literature on the “inverted classroom model”. Accordingly, high student satisfaction and a significant learning success were achieved. The question as to whether this concept is fundamentally superior to traditional teaching methods cannot be unequivocally answered. However, the results of our study suggest that the classification of periodontal patients (staging & grading) cannot be adequately taught in classroom-based lectures but requires innovative teaching methods, such as the ICM or case-based learning, which support procedural skills acquisition. 

Although the 2020 summer semester was a rather negative experience due to the COVID-19 pandemic, we gained a few positive insights. Probably, the pandemic has accelerated digitization in dental and medical education, and innovative teaching concepts like the ICM will have a formative influence on future curricula. 

## Authorship

Equal first authors: Marius Crome and Knut Adam.

## Competing interests

The authors declare that they have no competing interests. 

## Figures and Tables

**Table 1:**
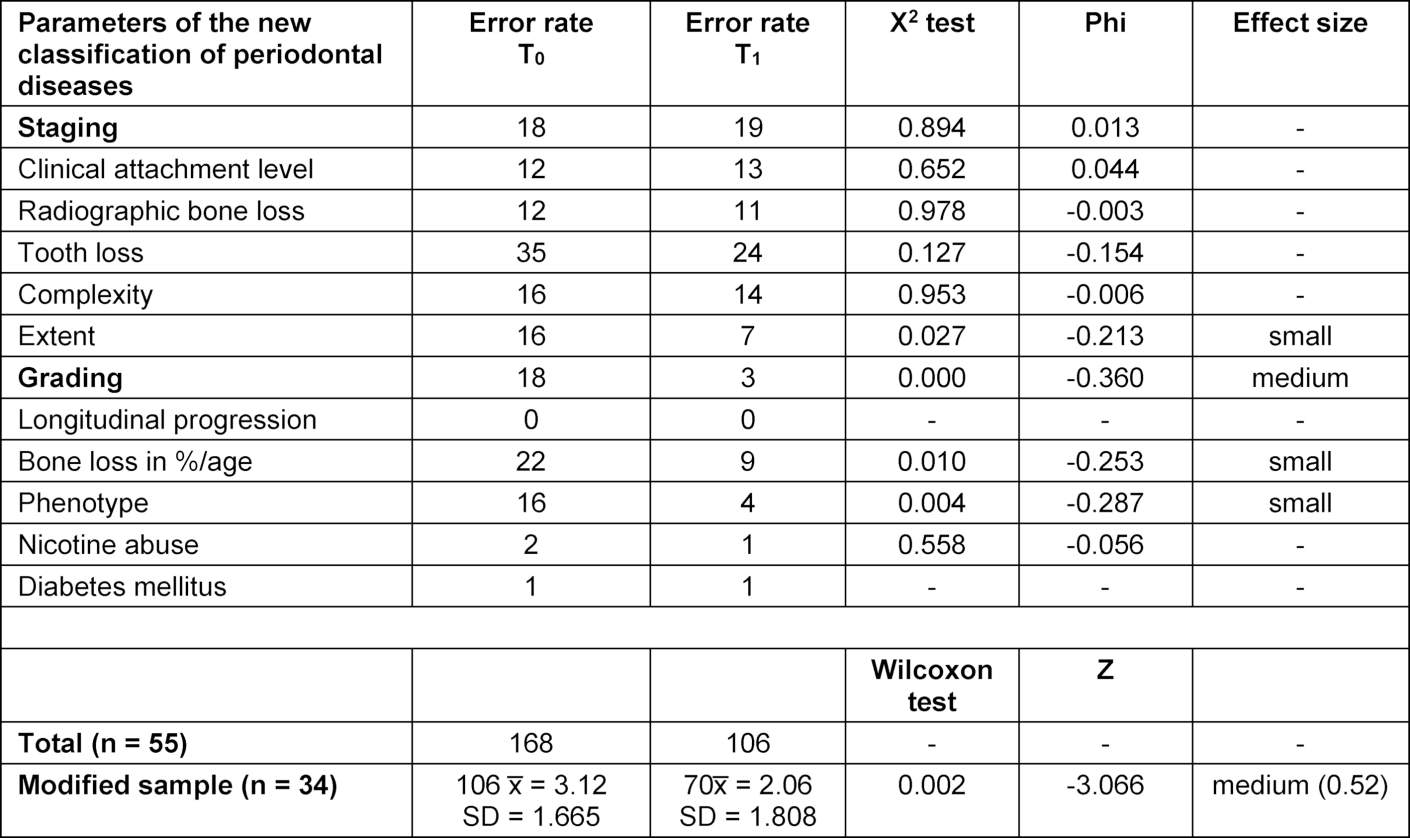
Statistical evaluation of learning success
